# Supplementary figures and images for: Wagers for work: Decomposing the costs of cognitive effort
Source: PLoS Comput Biol. 2024 Apr 29;20(4):e1012060. doi: 10.1371/journal.pcbi.1012060 (PMC11081491; doi:10.1371/journal.pcbi.1012060)

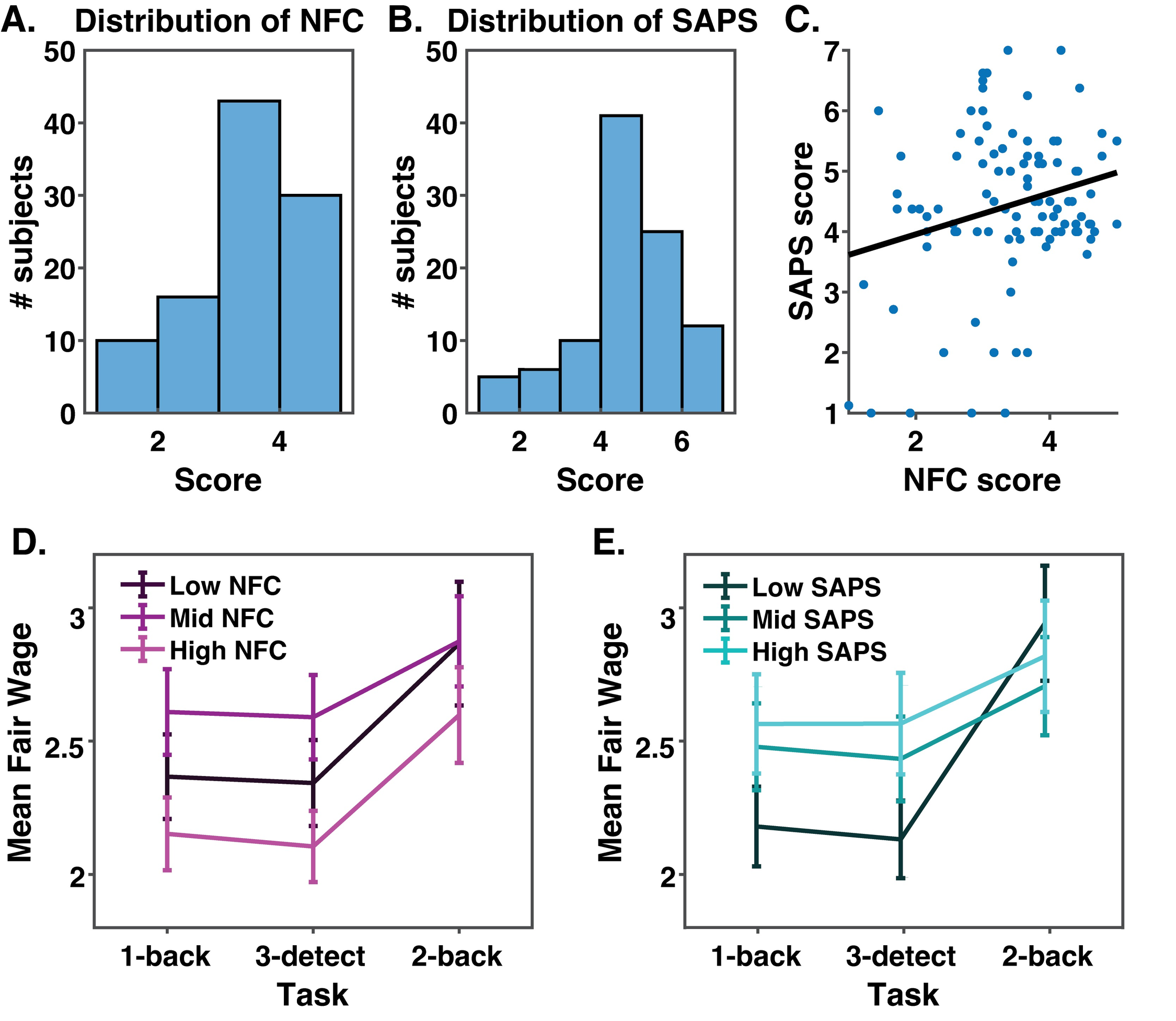

Supplement: S1 Fig — All subjects completed 10–11 ratings of each task, but only between 1 and 11 rounds per task. Here we plot the mean fair wage for the subjects who completed 1 to 11 iterations of each task, grouped by the total number of iterations they completed. Subjects who completed more task iterations are plotted in darker colors. This illustrates the diversity in fair wage ratings for each task across subjects, as well as the stability of the ratings subjects gave to each task. In addition, it shows that, due to the design of our task, subjects who asked for high fair wages on one of the tasks did indeed complete fewer iterations of that task. Error bars are drawn with standard error of the mean. (TIF) [file pcbi.1012060.s001.tif]

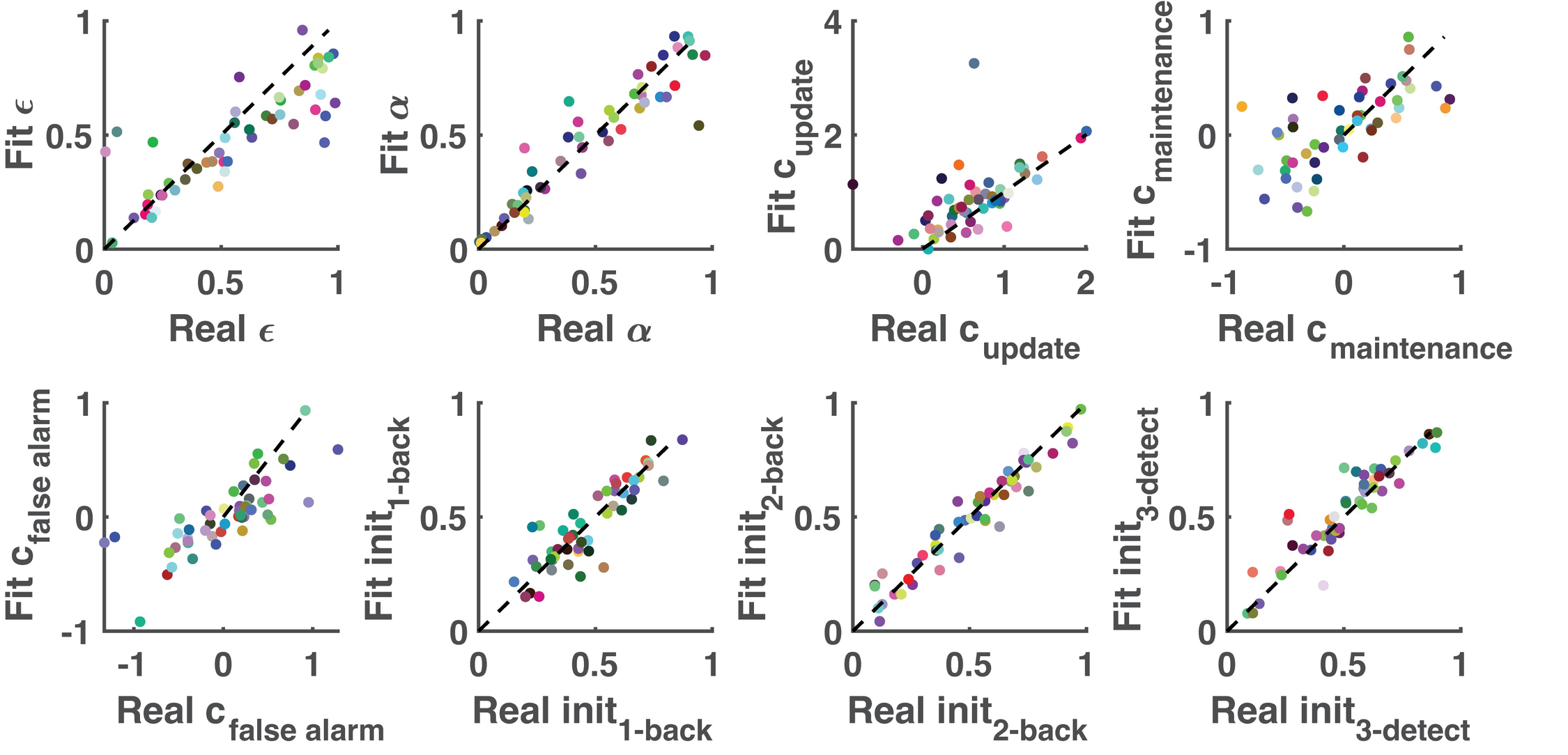

Supplement: S2 Fig — A. Distribution of Need for Cognition (NFC) scores within the experimental sample. Scores have been normalized by the number of questions answered such as not to lower the mean of the distribution artificially. The distribution of NFC scores in our sample is right-skewed compared to the typical distribution of NFC scores. B. Distribution of Short Almost Perfect Scale (SAPS) scores. Scores have been normalized by the number of questions answered such as not to artificially lower the mean of the distribution. The distribution of SAPS scores in our sample is typical of both in-person samples and other samples on MTurk. C. NFC scores versus SAPS scores. NFC and SAPS scores were positively correlated (r = 0.24; p < 0.01). D. Mean fair wage rating on the 1-back, 3-detect, and 2-back tasks by tertile split NFC groups. Error bars were drawn using the standard error of the mean (SEM). There was a significant quadratic relationship of NFC and mean fair wage ratings (β = -0.03). Post-hoc t-tests confirmed that the significant quadratic effect of NFC was only driven by mid NFC subjects having significantly higher fair wage ratings than high NFC subjects (p < 0.01). E. Mean fair wage rating on the 1-back, 3-detect, and 2-back tasks by tertile split SAPS groups. Error bars were drawn using the SEM. A 3-way ANOVA, revealed no effect of SAPS group (F = 2.2, p > 0.05) or of the interaction of SAPS group and task identity (F = 1.5, p > 0.05) on fair wages. (TIF) [file pcbi.1012060.s002.tif]

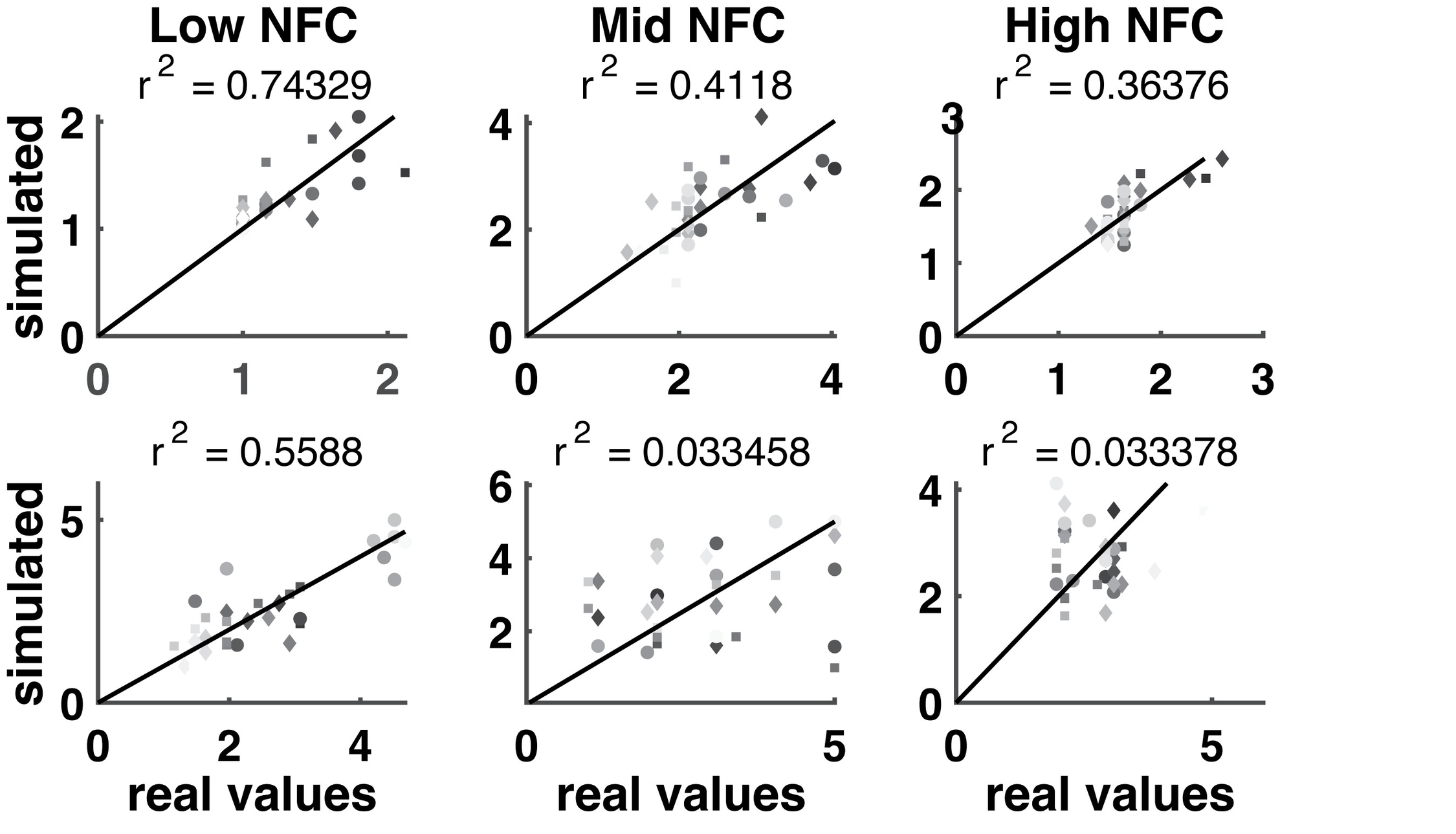

Supplement: S3 Fig — For each subject, we simulated data using the model with the highest model responsibility for that subject, and their fit parameter values. Here we have selected 2 random subjects from each NFC tertile (left: low NFC, middle: middle NFC, right: high NFC) and plotted their real and fit fair wage values. The title of each plot is the mean r-squared value after 100 simulations with the subject’s best fit model and best fit parameter values. Markers are shaded such that later trials are displayed in darker colors, and the shape of the marker indicates which task was rated (squares are 1-back ratings, circles are 3-detect ratings, and diamonds are 2-back ratings). (TIF) [file pcbi.1012060.s003.tif]

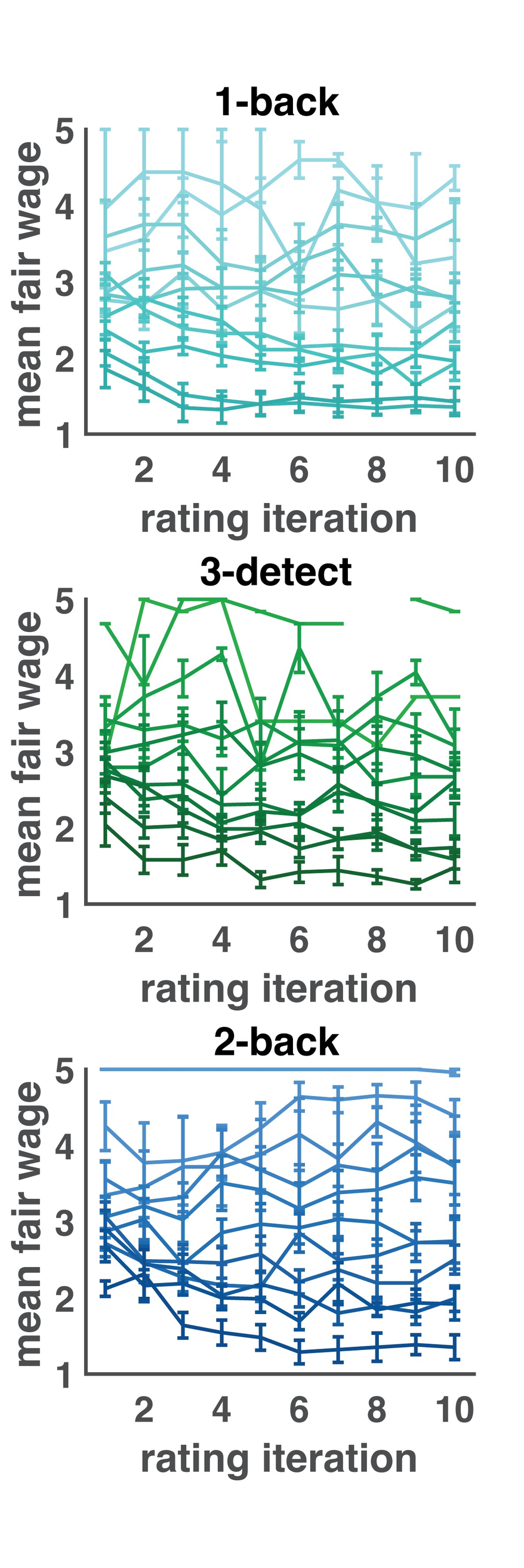

Supplement: S4 Fig — A dataset of simulated subjects was produced with random parameter values (constrained by the bounds of those parameters), and then fit with the same procedure as real subject data. Here we show the fits for 50 subjects out of 100, where each subject’s fits are plotted in a unique color. The identity line is overlaid on each subplot in black. Comparing the fit parameter values to the real values reveals the high fidelity of the model fitting procedure. Models were fitted using the Computational Behavioral Modeling (cbm) toolbox of Piray et al (2019). All candidate models were visually inspected and verified as recoverable to avoid fitting models with parameter tradeoffs. Only models with parameter recoverability were fit to real subject data. (TIF) [file pcbi.1012060.s004.tif]
