# Supplementary material for: Wagers for work: Decomposing the costs of cognitive effort
Source: PLoS Comput Biol. 2024 Apr 29;20(4):e1012060. doi: 10.1371/journal.pcbi.1012060 (PMC11081491; doi:10.1371/journal.pcbi.1012060)
Supplement: S1 Text — (DOCX) [file pcbi.1012060.s005.docx]

**_­­­­­_S1 Text**

*Covariance between cost components, but not between cost parameters*

Because the 2-back was associated with the most WM updating, most errors, and most interference in WM, many of the cognitive components of task completion that came out of our process model were highly correlated. For example, across all subjects & task rounds, updates, maintenance, lures, and false alarms were all correlated (interference vs. false alarms r = 0.48, p < 0.001; interference vs. maintenance r = 0.53, p < 0.001; maintenance vs. false alarms r = 0.31, p < 0.001; updates vs. maintenance r = 0.86, p < 0.001; updates vs. false alarms r = 0.43, p < 0.001; updates vs. interference r = 0.62, p < 0.001). In addition, when we ran within-subject correlations of these components across rounds, they were significantly correlated within 91% (maintenance vs. interference), 44% (maintenance vs. false alarms), 74% (interference vs. false alarms), 100% (updates vs. maintenance), 66% (updates vs. false alarms), and 90% (updates vs. interference) of subjects. One consequence of this may be that the cost parameter values associated with these components trade off with one another in model fitting, artificially raising or lowering each other. In addition, model selection may have been impacted, resulting in a low number of subjects who were best fit by models including multiple costs. Because most subjects’ data are best captured by a model including only one cost of cognitive effort, one might wonder whether the cost parameters obtained from our models are capturing one cost only, but incorrectly assigning them to three different components due to the relatedness of the components. To compare cost parameter magnitudes across models including only one or two cost parameters each and to ensure their separability, we constructed joint posterior distributions over parameter values using the outputs of the CBM toolbox [[1]](https://paperpile.com/c/Ilrqru/pgVVS). We also examined whether these parameter values traded off during model fitting by examining their covariances, which are derived from the inverse Hessian of the search gradient within the multidimensional parameter space.

The way we constructed these joint posterior distributions, as they were not included in the outputs of the CBM toolbox, was by calculating a within-subject posterior distribution over the three cost parameters of interest $\theta_{0}, \theta_{1}, \theta_{2}:P(\theta_{0}, \theta_{1}, \theta_{2}|D^{1})$ where $D^{1}$is the data from subject 1 (more details on this procedure are available in the Methods section). We constructed this distribution over all models, even those which did not include all parameters of interest, by instead using the marginal distribution of those parameters from all the models which did contain them (effectively, a prior). For example, if $\theta_{2}$ is not included in model 1, then the prior over$\theta_{2}$, drawn from all the other models, is $P(\theta_{2}|D^{1}, m\neq1)$. This allows for self-consistent marginal distributions over the individual parameters. However, we acknowledge that this is not the only way that we could have chosen to compute the joint posterior distribution, and this choice may have an impact on the overall correlations between the parameters.

First, there was no evidence that these single cost parameters somehow capture just one underlying component, rather than 3 separate ones, as the posterior distributions over their magnitudes are mostly non-overlapping on the group level (Fig 3B). While the update and interference costs are of similar magnitudes, and therefore overlapping, the negligible covariance between update and interference costs suggests that they did not trade off in model fitting.

Second, the covariances between update, maintenance, and interference costs, which were all present in the most prevalent model that included multiple costs, were all within an acceptable range. The covariance between the update and maintenance costs was largest, at 0.22. Between the update and interference costs, the covariance was 0.0492. Between the interference and maintenance costs, it was -0.0763. Remember that these covariances are influenced both by the empirical covariances between fit parameter values as well as their covariances during parameter fitting. And in fact, we also verified in a pre-model fitting generate and recover procedure that individual cost parameters were being accurately fit even in models with multiple costs (S4 Fig).

**References**

1. [Piray P, Dezfouli A, Heskes T, Frank MJ, Daw ND. Hierarchical Bayesian inference for concurrent model fitting and comparison for group studies. PLoS Comput Biol. 2019;15: e1007043.](http://paperpile.com/b/Ilrqru/pgVVS)
