# Supplementary material for: Wagers for work: Decomposing the costs of cognitive effort
Source: PLoS Comput Biol. 2024 Apr 29;20(4):e1012060. doi: 10.1371/journal.pcbi.1012060 (PMC11081491; doi:10.1371/journal.pcbi.1012060)
Supplement: S2 Text — (DOCX) [file pcbi.1012060.s006.docx]

**S2 Text**

*Supplementary model fits and analyses*

*Joint cost-learning and cost-changing mechanisms*

98 of 100 subjects were best fit by cost-learning ɑ-only models which did not include dynamically changing costs of cognition. The lack of a cost-changing process in 98% of subjects was surprising, due to the many studies demonstrating that prolonged cognitive work reduces subjects’ willingness and ability to continue such work [[1–3]](https://paperpile.com/c/6VnjGB/3Uqme+ww2wm+9c4XN). To confirm the validity of these findings, we ran a supplementary hierarchical model fit, with the intent of comparing the winning 𝛼-only class of models to a joint 𝛼-δ class of models which included both linear and quadratic δ cost-changing terms (see Methods). This hierarchical model fit did not include the original linear δ-only class of models. It included 112 models, ⅓ of which were the original ɑ-only models, ⅓ were linear 𝛼-δ models (modeling a linear cost-changing process due to fatigue or task practice, in tandem with an incremental cost-learning process), and the remainder being quadratic 𝛼-δ models (modeling a quadratic cost-changing process and an incremental cost-learning process).

These new alpha-delta models took on 1.3e-6% of model frequency in this subject pool, essentially 0%. Though we did not compare them directly via joint inclusion in the hierarchical model fitting procedure, for comparison the linear cost-changing-only models from our original formulation took on 2.36% of model frequency. This provides further evidence of the low prevalence of cost-changing dynamics in the fair wage ratings we report here.

*Testing a different process model*

We defined a “lure” trial as any trial on the 2-back task in which the stimulus 1 trial back in working memory storage matched the stimulus currently on screen. We hypothesized that these lures would evoke interference, leading to a slightly higher probability of false alarm errors as well as greater feelings of subjective effort from having to protect against this interference. However, a different, more expansive definition of lures is possible. For example, the same type of trial could also be considered a lure on the 3-detect task, if the subject has had a lapse in memory and cannot recall whether the current stimulus constitutes the 2nd or 3rd stimulus of the same type. To assess this possibility, we ran a series of analyses, first assessing false alarm responding, and second comparing process models according to their relative complexity-controlled model fits.

We hypothesized that lures should be associated with at least a slightly greater likelihood of false alarm responding. Subjects committed the greatest number of false alarm errors during the 2-back task, from 0 to 9 per round. They committed on average 1.8 per round (std 1.5). By contrast, they committed virtually no false alarm errors during the 3-detect task (average 0.3, std 0.73). This suggests that, at very least, putative “lure” trials do not elicit the same degree of interference during the 3-detect task as during the 2-back task.

Next, we ran model comparison to determine which definition of lures provided a better complexity-controlled fit to subject data. We therefore coded lures as occurring during the 2-back only, and separately as occurring during both the 2-back and 3-detect tasks (checking that the associated parameters were suitably recoverable). We then fit the resulting models to subject data. Because every cost parameter we add to the models increases computational time by tens of hours (due to the search we perform over all models containing all combinations of costs; see Methods), we ran a more limited search over the 30 simplest models, instead of assessing all the recoverable models possible (over 100). We found that models containing the more expansive definition of lures had model frequencies of 7e-8% - essentially, 0%. By contrast, the models containing the 2-back-only lures exhibited 34% model frequency. That is, there was no evidence that a more expansive definition of lures accounts for any fair wage ratings.

**References**

1. [Friese M, Loschelder DD, Gieseler K, Frankenbach J, Inzlicht M. Is Ego Depletion Real? An Analysis of Arguments. Pers Soc Psychol Rev. 2019;23: 107–131.](http://paperpile.com/b/6VnjGB/3Uqme)

2. [Muraven M, Baumeister RF. Self-regulation and depletion of limited resources: does self-control resemble a muscle? Psychol Bull. 2000;126: 247–259.](http://paperpile.com/b/6VnjGB/ww2wm)

3. [Wiehler A, Branzoli F, Adanyeguh I, Mochel F, Pessiglione M. A neuro-metabolic account of why daylong cognitive work alters the control of economic decisions. Curr Biol. 2022;32: 3564–3575.e5.](http://paperpile.com/b/6VnjGB/9c4XN)
